# Differential response to mosquito host sex and parasite dosage suggest mixed dispersal strategies in the parasite *Ascogregarina taiwanensis*

**DOI:** 10.1371/journal.pone.0184573

**Published:** 2017-09-13

**Authors:** John Soghigian, Todd Livdahl

**Affiliations:** Department of Biology, Clark University, Worcester, MA, United States of America; Swedish University of Agricultural Sciences, SWEDEN

## Abstract

Mixed dispersal strategies are a form of bet hedging in which a species or population utilizes different dispersal strategies dependent upon biotic or abiotic conditions. Here we provide an example of a mixed dispersal strategy in the *Aedes albopictus* / *Ascogregarina taiwanensis* host/parasite system, wherein upon host emergence, the gregarine parasite is either carried with an adult mosquito leaving the larval habitat, or released back into the larval habitat. We show that the parasite invests a larger proportion of its dispersing (oocyst) life stage into adult female mosquitoes as opposed to adult male mosquitoes at low parasite exposure levels. However, as the exposure level of parasite increases, so does the parasite investment in adult males, whereas there is no change in the proportion of oocysts in the adult female, regardless of dose. Thus, *A*. *taiwanensis* is utilizing several dispersal strategies, depending upon host sex and intraspecific density. Furthermore, we demonstrate that this parasite reduces body size, increases time to emergence in females, and leads to a reduction in estimates of per capita growth rate of the host.

## Introduction

Mixed dispersal strategies are a form of bet hedging in which a species or population utilizes different dispersal strategies dependent upon biotic or abiotic conditions. Dispersal strategies are recognized both theoretically and empirically as consequentially reducing negative interactions between and among species [1–3] and reducing risk due to spatial or temporal stochasticity in variable environments [4]. However, dispersal carries with it an inherit risk, as there is no guarantee of finding a suitable habitat for dispersing individuals. Mixed dispersal strategies can help alleviate the risk, such as in *Gerris* water striders where winged morphs may disperse in the event of habitat desiccation but have lower fecundity compared to wingless individuals of the same populations [5–9]. Mixed dispersal strategies may also alleviate the effects of density, as in the case of wing-dimorphic plant hoppers (Homoptera: Delphacidae). Winged morphs can disperse up to hundreds of kilometers [10,11]. When invading temporary habitats, the first generation of plant hoppers is almost always wingless; however, subsequent generations have winged forms in temporary habitats as food resources decline and density increases [12].

Mixed dispersal strategies are widespread. In microsporidian *Amblyospora* species, which infect a wide range of genera within the mosquito family Culicidae [13], infection by some species in female host mosquitoes leads to vertical transmission and is relatively benign to female larvae; in males, infection leads to larval mortality and release of spores into the larval habitat [14]. The microsporidian parasite responds to the “environmental” condition of host sexuality and alters its dispersal strategy. In the case of mosquitoes, adult flight has very different consequences for parasites within adult females, which will return to larval habitats for oviposition, versus males, which are unlikely to visit another larval habitat during their lifetime. Fellous and Koella [15] showed that *Ascogregarina culicis* (Apicomplexa: Lecudinidae), a parasite of the mosquito *Aedes aegypti*, favors one transmission route in the male host, while it favors another in the female host, similar to microsporidians. In male hosts, *A*. *culicis* localizes within the host such that the majority of its oocysts, the infectious stage of the parasite, are released into the larval habitat in the pupal exuviae, favoring local transmission. In female hosts, the parasite localizes in the emerging adult, which favors a dispersal strategy. Furthermore, the authors found a unidirectional effect of parasite density: as the mosquito is exposed to higher levels of parasites, localization of the parasite in both males and females trends more toward a dispersal strategy [15]. That some *A*. *culicis* oocysts remain in the emerged adult mosquito, while others are released directly into the larval habitat, represents a case in which two distinct dispersal strategies present for this species of *Ascogregarina*, and result from the interaction of *Aedes* host and *Ascogregarina* parasite life cycle.

*Aedes* mosquitoes are notable for their propensity to transmit a number of major human diseases [16–18] and are a cosmopolitan genus of mosquitoes with diverse habitat preferences. Within the genus are container-dwelling mosquitoes, such as *Ae*. *aegypti* and *Ae*. *albopictus*, which utilize temporary or semi-permanent container habitats, both natural and domestic, for immature life stages [19]. While adult female mosquitoes may return to the same container or seek out a new habitat, male mosquitoes have no reason to return to container habitats as adults. Within these container habitats, *Aedes* mosquitoes are frequently parasitized by the Apicomplexan gregarine parasites within the genus *Ascogregarina* [20]. Considered the most invasive vector species of today, *Aedes albopictus* has spread from a home range in Asia to every continent [21]. Due to the success of the invasive host mosquito, *Aedes albopictus* and *Ascogregarina taiwanensis* are perhaps the best studied host/gregarine system [22–28]. *Ascogregarina taiwanensis* has been found infecting its host throughout much of *Ae*. *albopictus*’ natural [29] and invasive ranges (in North America [22], in Brazil [30], and in Bermuda [31]).

The life cycle of *Ascogregarina* parasites is best characterized from *A*. *taiwanensis* infecting *Ae*. *albopictus* [20]. Infection by *Ascogregarina* begins when filter feeding larvae of any instar ingest oocysts of the parasite; these oocysts release sporozoites into the larval midgut [32]. *Ascogregarina taiwanensis* develops intracellularly within its host until the release of a host molting hormone, at which point trophozoites of the parasite migrate to the host malphigian tubules for sexual reproduction [23]. Sexual reproduction occurs there through syzygy [24] and resulting oocysts remain in the host until eclosion; some parasites are released back into the larval environment upon emergence, while others are carried with adults and released during defecation or oviposition [29]. Virulence within natural hosts appears to be so low that some authors have suggested that these parasites are largely benign [27,29,33], and a recent study has shown that *Ascogregarina* may alter larval feeding behaviors and reduce risk of predation on their hosts [34]. However, environmental factors such as food availability and dosage of parasites can lead to elevated mortality rates and developmental delay in aquatic stages of the mosquito and reduced body size in adults [35–37].

Density-dependent shifts in dispersal strategy by a parasite may be a result of crowding within hosts which could be detectable by a decrease in the per-capita rate of change of the parasite. Further, distinct dispersal strategies for males and females within mosquito host/parasite systems may be the outcome of selection on the parasite to avoid adult males, or, it is possible that these distinct dispersal strategies are instead a result of components of male life history traits. Males develop faster and have smaller body size relative to females, and it is possible that these features create a mechanical dispersal limitation for the parasite. If so, parasite loading in adult mosquitoes would vary based on body size or time to emergence, regardless of sex.

Here we consider whether mixed dispersal strategies are present in *A*. *taiwanensis*, and whether they might result from components of individual host fitness, such as body size and time to emergence, or from sex of the mosquito host. Our hypothesis is that *A*. *taiwanensis* employs a mixed dispersal strategy, varying with host sex, and that this mixed dispersal strategy will vary with parasite dosage. Despite the wealth of studies on the effect of *A*. *taiwanensis* in its host, virtually all such research between host and parasite has focused on the parasite’s effect on the host, and has not considered aspects of parasite life cycle completion. We vary the density of parasites within a host by applying several different dosages of parasites, and we examine components of parasite and host life history to generate fitness proxies for both host and parasite. Under the hypothesis that *A*. *taiwanensis* is a parasite and that it will exhibit effects of density dependence, we expect to see fitness consequences on its host that will be detectable via declines in fitness proxies and survival as dosage increases, and a concomitant decline in the parasite growth rate as dosage increases. Through these estimates, we can evaluate both the fitness consequence of the parasite on its host, as well as the success of the parasite under increasing parasite density, while simultaneously testing for multiple dispersal strategies.

## Methods

### Collection and culturing of Aedes albopictus and Ascogregarina taiwanensis

We established a colony of *Aedes albopictus* from field-collected ovitrap substrates provided by the Bermuda Ministry of Health. Prior to hatching, we rinsed egg slats with distilled water, as field populations of *Ae*. *albopictus* in Bermuda are infected with *A*. *taiwanensis*. We hatched egg slats in 1g/L of nutrient broth (Difco Labs), removed first instar larvae after 24 h and reared them on powdered yeast. Emerged adults were placed in cages with oviposition containers and sugar wicks with 10% sucrose solution and offered a blood meal via a Hemotek membrane feeding system (Blackburn, UK) containing sheep blood once every two weeks. We maintained this colony in an insectary at 24C and 80% RH, with 16:8 (L:D) h.

We collected *A*. *taiwanensis* in Bermuda under the supervision of the Department of Vector Control, Bermuda Ministry of Health, as reported in Erthal et al. [28]; no permits were required for this species, as this parasite is not a protected species and collections took place under supervision of Vector Control personnel. We held pupae in a vial, causing adult mosquitoes to emerge and die. We then returned the resulting homogenate to the United States. We had previously confirmed that the *Ascogregarina* parasite in Bermuda was *A*. *taiwanensis* [28]. We passed the parasite through three generations of *Ae*. *albopictus* hosts from Bermuda prior to this study, amplifying the parasite by collecting parasite material from dead host homogenate. Following death of all adults, we filtered the oocysts with a nylon coffee filter to remove large mosquito parts, then determined the concentration of oocysts using a hemocytometer.

### Experimental design

To mimic low host density, high-resource conditions, we established microcosms that contained 50 ml of dechlorinated H_2_0, 15 *Ae*. *albopictus* larvae, and one of four parasite levels: 0, 100 oocysts/ml, 1,000 oocysts/ml, or 10,000 oocysts/ml. We replicated each parasite exposure level six times, and stored the microcosms in our insectary. We fed microcosms with 0.05g of powdered brewer’s yeast every 2 d. Each microcosm was checked daily and any pupae were individually rinsed and transferred into a 1.5 ml centrifuge tube containing 500 μl of dechlorinated H_2_0. Upon emergence, adults were transferred into a separate centrifuge tube. We measured the left wing from each adult and recorded wing length and date of emergence.

#### Per capita growth rate of the host

We used wing lengths and emergence dates of mosquitoes to estimate the per capita rate of change, r’, for each microcosm replicate following Livdahl and Sugihara [38]:

r′=ln⁡[(1N0)∑xAxf(Wx)]D+[∑xxAxf(Wx)∑xAxf(Wx)] (1)

where N_0_ is the initial number of females in the replicate (assumed to be 50% of the cohort), A_x_ is the number of females emerging on day x, f(W_x_) is the linear regression that estimates the number of eggs produced by a female on day x with wing length W, and D is the number of days from eclosion to oviposition. We assumed D to be 14 days [39]. For f(W_x_), we used the previously published wing length regression for *Ae*. *albopictus* from Lounibos et al. (*r*^2^ = 0.713, *N* = 91, *P* < 0.001) [40]:

$$f(w_{x})=78.02w_{x}-121.240$$ (2)

#### Per capita growth rate of the parasite

For half of the replicates per dosage treatment, chosen at random, we also quantified parasite success. Following wing measurements, the adult was homogenized in 500 μl of H_2_0 using a hand pestle. We quantified oocysts released during emergence in pupal exuviae as well as homogenized adult tissues using a hemocytometer, and used these counts to estimate the per capita growth rate of the parasite at different dosages. Because the generation time of this parasite corresponds closely with the generation time of its host, this parasite does not fit into traditional microparasitic modeling and thus we chose to take generation time into account:

rp=ln⁡[∑xOocsytst,x/Oocystse]∑xxOocsytst,x∑xOocsytst,x (3)

Assuming that oocysts in the adult mosquito and pupal exuviae (= Oocysts_t_) scales linearly with oocyst production per host generation, and that the number of oocysts consumed by mosquitoes in the replicate (= Oocysts_e_) can be measured by subtracting the number of oocysts in the microcosm originally from the number remaining, r_p_ should approximate the parasite’s per capita growth rate. On the other hand, it is possible that oocysts remaining in the adult male will have no opportunity for reproduction, as adult male mosquitoes are not thought to return to larval habitats. In this case, an estimate of *r* for the parasite that includes oocysts leaving the larval habitat in adult males may be biased; thus, we also consider a version of r_p_, r_m_, wherein the oocysts in the adult male are excluded from the summed total of oocysts in the adult mosquito and pupal exuviae (= Oocyst_t_).

We assessed the remaining oocysts in the microcosm by taking one ml samples from each replicate. We then centrifuged all water samples at 15,000g for 5 minutes. Following this, we removed the top 900 μl of water, then vortexed the remaining 100 μl to mix. We then quantified any remaining oocysts with a hemocytometer, and then extracted DNA from each tube. We also attempted to amplify any parasite DNA within each tube [28]. Because we attempted to assess the remaining oocysts in each microcosm and found that we could detect none remaining through visually checking of samples with a hemocytometer or through extracting DNA and amplifying any parasite DNA following Erthal et al. [28], we assumed that all oocysts in the microcosm had been consumed by the hosts, and treated Oocysts_e_ as equal to the starting density of oocysts.

#### Statistical analyses

All statistical analyses were performed in R version 3.2 [41].

For all microcosms, we used ANOVA to explore the effect of our explanatory variable, parasite dosage, on response variables of per capita rate of increase for host and parasite (r’ for the host, r_p_ and r_m_ for the parasite), survival for both sexes, sex ratio of surviving mosquitoes, mean wing length, and mean time to emergence. We used the R function Anova from the R package car for summarizing model effects [42]. We tested model residuals using the Shapiro-Wilk test and the R function shapiro.test, while we tested assumptions of homogeneity of variances using Levene’s Test [42]. If a model violated any assumptions, we used a randomization ANOVA following Mitchell and Bergmann in which a null distribution is generated via randomization [43]. For ANOVA models that had significant or nearly significant effects or effects, we calculated effect sizes using partial η^2^ in the lsr package [44], and examined pairwise differences between factor levels using Tukey’s Honestly Significant Differences as implemented in the TukeyHSD function from the base R stats package [40].

To assess parasite success within individual male and female mosquitoes, we used ANCOVA with the response variables of total oocysts (combining the oocysts in the pupal exuviae with those from the adult–Oocysts_t_, above) or percent of oocysts in the adult (out of total oocysts counted) and explanatory variables of sex and parasite dosage, with a covariate of wing length and time to emergence, and a random effect for the microcosm from which the individual emerged. For random effects models, we used the lmer function from the package lme4 [45]. We evaluated assumptions as above, and we used the same randomization ANOVA technique as above in the event of assumption violations. We used the lsmeans package [46] to evaluate significant differences between pairs of factors while accounting for both covariates and the random effect.

## Results

### ANOVA/ANCOVA model assumptions

Our ANOVA and ANCOVA models met assumptions of normality and homogeneity of variances (P values > 0.05), save for three. Our ANCOVA model of total oocysts in the mosquito and the explanatory variables of sex and parasite dosage rejected homogeneity of variances, while our ANOVA models for response variables of wing length and time to emergence both rejected normality of residuals (P<0.01 in all cases). For these models, we used a randomization ANOVA to evaluate significant effects.

### Parasite effects on the host

**Fig 1:**
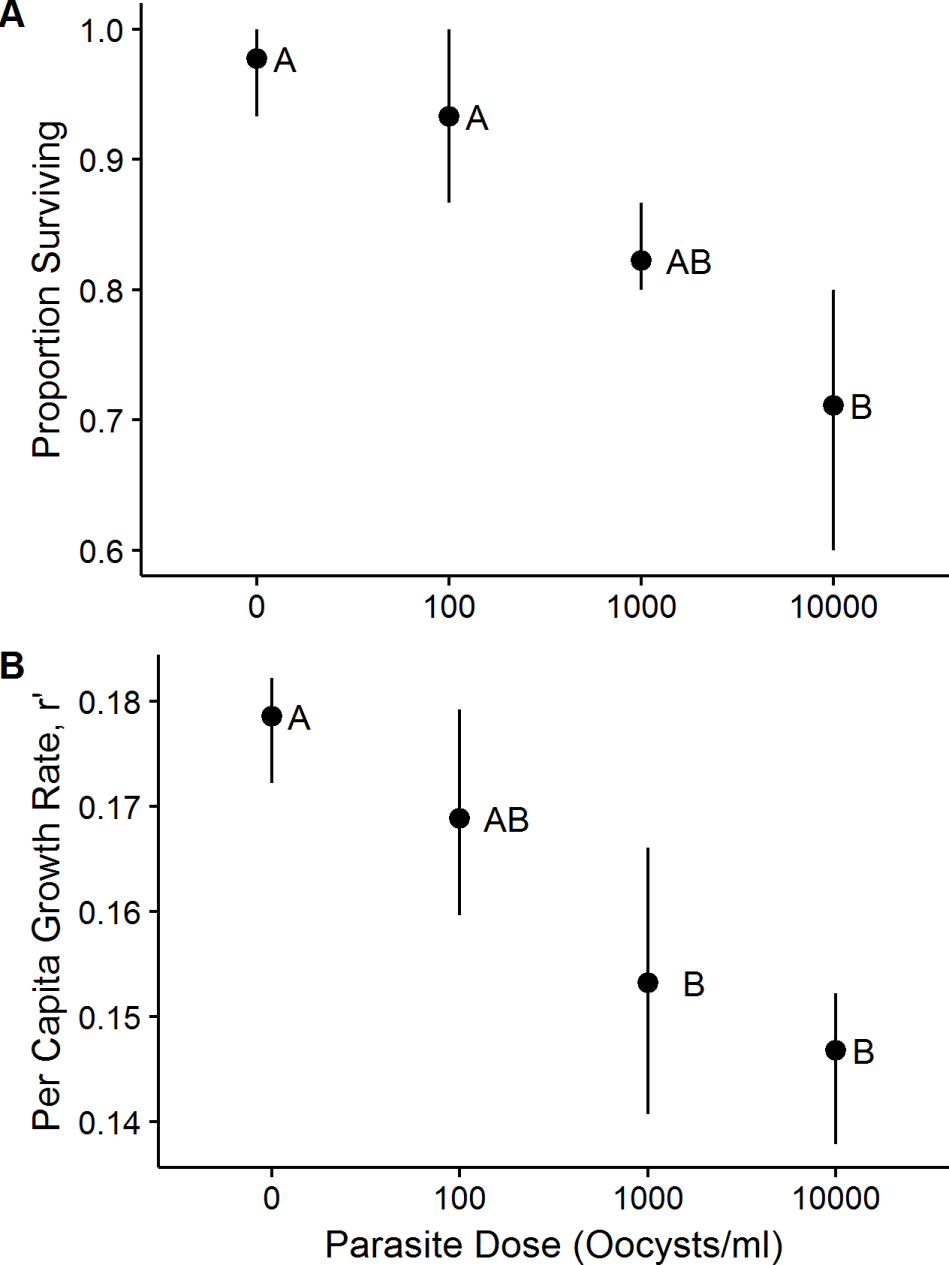
The mean proportion of mosquitoes surviving to adulthood (A) and mosquito per capita rate of increase (B) exposed to different parasite dosages. Host *Aedes albopictus* fitness declines by two measures as dosage of *Ascogregarina taiwanensis* increases. Different letters within each plot are significantly different based on Tukey’s HSD. Whiskers are bootstrapped 95% confidence intervals (1000 replicates).

**Fig 2:**
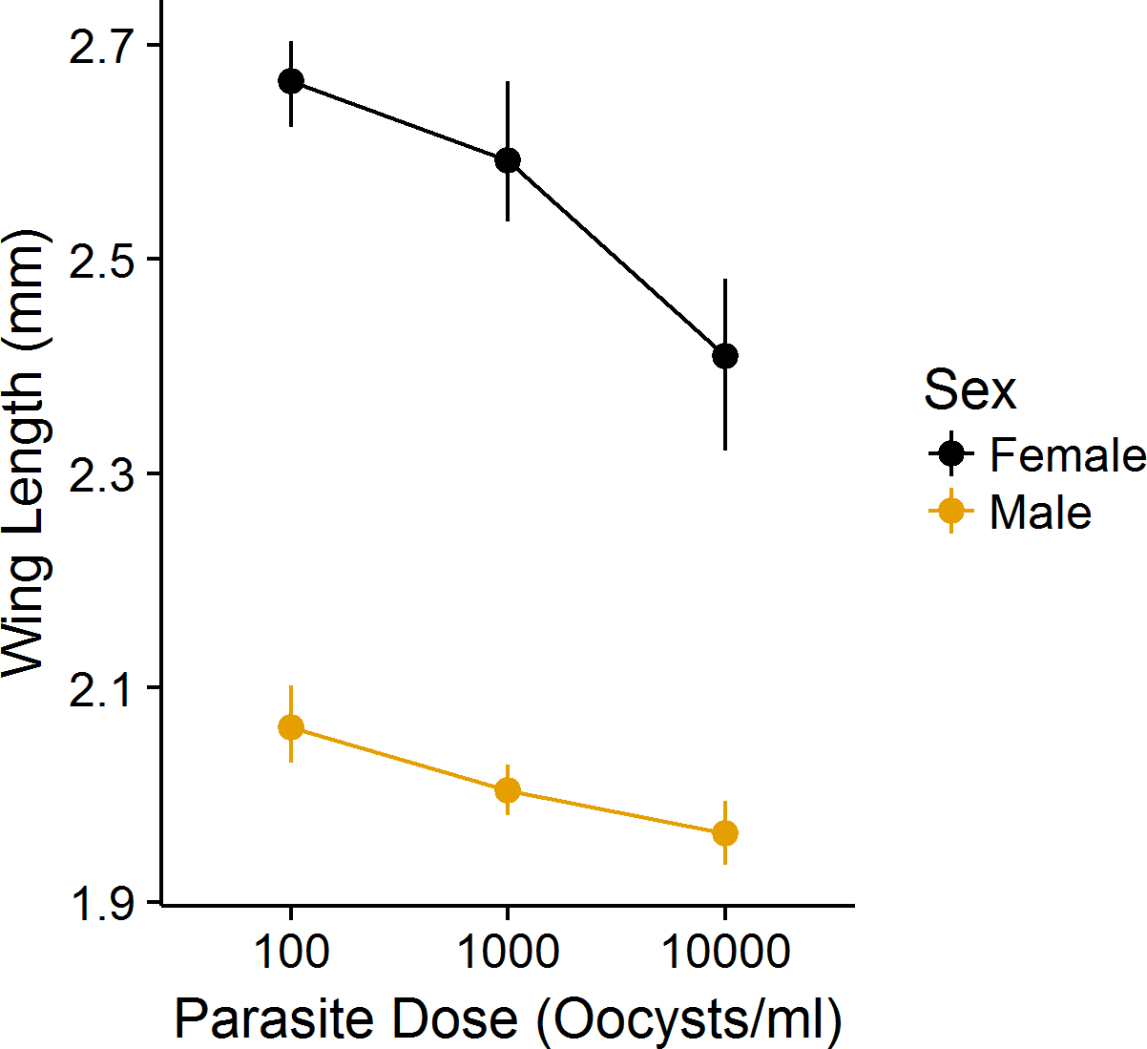
The mean wing length of female and male mosquitoes exposed to different parasite dosages. Both female and male mosquitoes show smaller body size when parasitized, but the relative effect for female *Ae*. *albopictus* appears greater, as evidenced by the interaction between sex and dosage (Table 2). Whiskers are bootstrapped 95% confidence intervals (1000 replicates); no confidence intervals for any points overlap.

We found that parasite exposure significantly decreased both survival (Fig 1A; F_3,8_ = 9.64, P<0.01; Tables A & D in S1 File) and host per capita rate of increase (Fig 1B; F_3,8_ = 9.07, P<0.01; Tables A & E in S1 File) in microcosms, and although this effect was only significant at high dosages (Fig 1), there was a large effect size associated with parasite dosage (Table 1; partial η^2^ for proportion surviving: 0.78; partial η^2^ for r’:0.73). We found no effect of dosage on mean sex ratio of host (Table A in S1 File; F_3,8_ = 0.04, P>0.95), nor on time to emergence of individual mosquitoes (Table B in S1 File; F_2,105_ = 0.32, P>0.73), although we found an effect of sex on time to emergence (F_2,105_ = 45.40,P<0.01) as expected. We found a significant effect of parasite dosage on wing length, and the effect of dosage depended on dosage level and sex (Fig 2; Table 2).

**Table 1:** Summary of partial ANOVA results for the explanatory variable of parasite dosage and some response variables**.

Response Variable	D.f.	Partial η^2^	F	P
Survival	3	0.78	9.64	<0.01*
r'	2	0.73	7.2	0.011*
r_p_	2	0.90	27.11	<0.01*
r_m_	2	0.88	22.75	<0.01*

* Significant at a 0.05 alpha level.

** Full ANOVA models and Tukey’s pairwise comparisons are in the supplemental materials.

**Table 2:** Randomization ANOVA results for the response variable of wing length and the explanatory variables of sex and parasite dosage.

Effect	D.f.	Partial η^2^	F	P
Dosage	2	0.32	27.85	<0.01*
Sex	1	0.87	687.16	<0.01*
Dosage:Sex	2	0.09	5.29	<0.01*
Residuals				

* Significant at a 0.05 alpha level.

### Parasite success in microcosms

**Fig 3:**
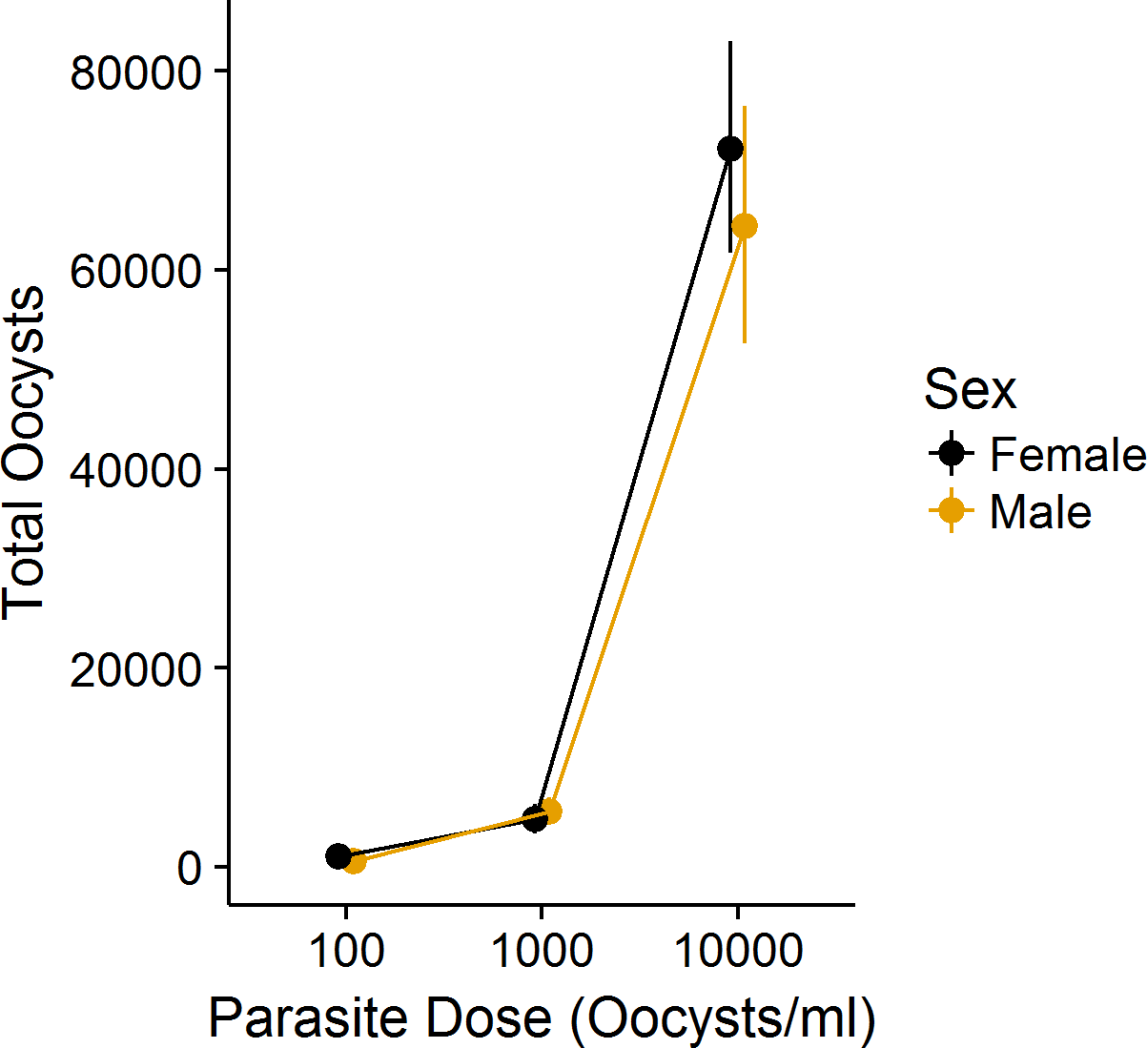
The mean total oocysts produced by mosquitoes exposed to different parasite dosages. Oocyst production within microcosms increases with dosage of parasite. Total oocysts produced did not significantly vary with sex (Table C in S1 File). Both sexes received equal doses; mean values are ‘dodged’ at each parasite dosage for visibility. Whiskers are bootstrapped 95% confidence intervals (1000 replicates).

**Fig 4:**
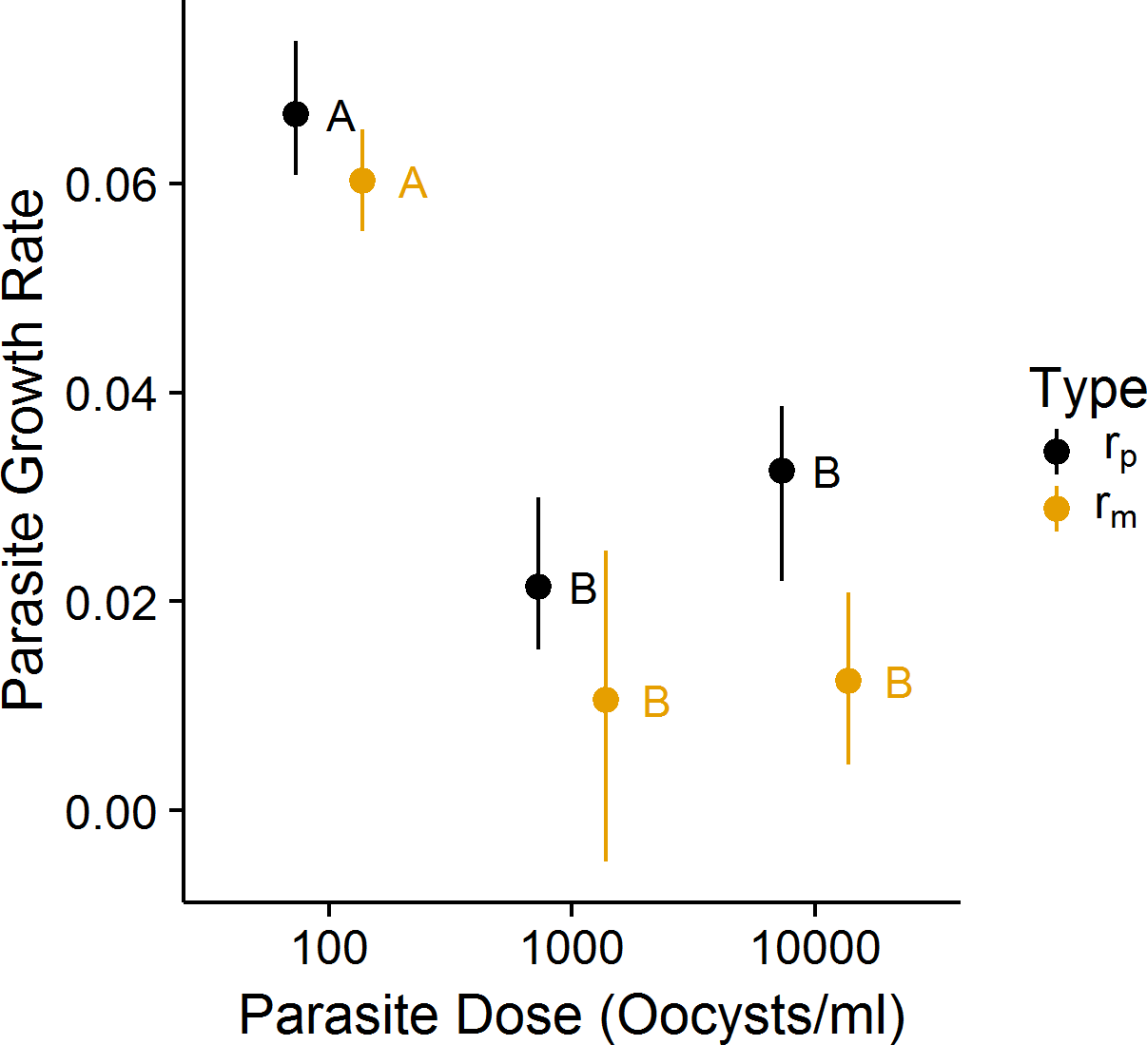
Two approximations of mean parasite per capita growth rate under different parasite doses to mosquito hosts. Parasite growth rate declined significantly regardless of which growth rate type, including oocysts from the male mosquito (r_p_) or not including them (r_m_) was used, suggesting an effect of density on parasite growth. Colored letters correspond to separate Tukey’s HSD tests on ANOVA models of each growth rate approximation. Whiskers are bootstrapped 95% confidence intervals (1000 replicates). Mean points are dodged for visibility.

**Fig 5:**
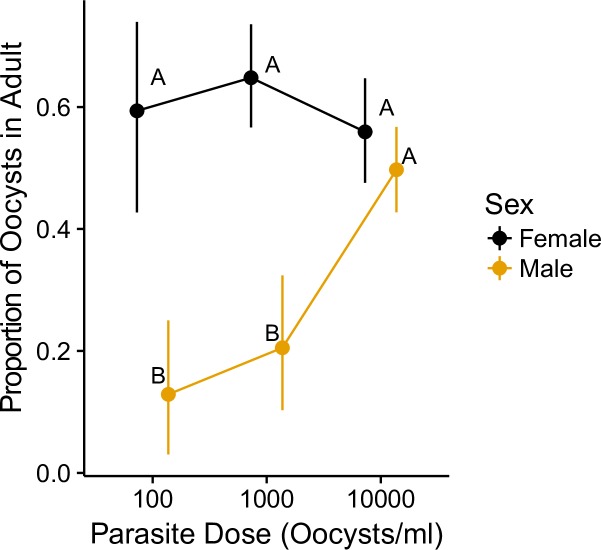
The percent of oocysts in the adult mosquito at three parasite dosages. Male mosquitoes had a smaller proportion of oocysts in the adult until the highest dosage, 10000 oocysts/ml, where male and female mosquitoes had the same percent of oocysts in adults. Different letters correspond to separate Tukey’s HSD tests on the ANCOVA model. Whiskers are bootstrapped 95% confidence intervals (1000 replicates). Mean points are dodged for visibility.

An increase in parasite dosage was related to an increase in the number of oocysts produced by hosts in microcosms (F_2,103_ = 290.16, P<0.01), regardless of host sex, wing length, or time to emergence of the adult (Fig 3, Table C in S1 File). Both our estimates of parasite per capita growth declined with parasite dosages above 100/ml (Table 1), although neither growth rate differed at the higher parasite doses of 1000/ml or 10,000/ml (Fig 4). Neither parasite growth rate suggested a significantly different relationship with parasite dosage. As expected, our parasite growth rate excluding adult male oocysts (r_m_) suggested a lower overall growth rate compared with r_p_, although only at the highest dosage did the confidence intervals for the two measures fail to overlap. We found a significant interaction in the proportion of oocysts in the adult between male and female mosquitoes (Fig 5; χ^2^_2_ = 11.39, P < 0.01); as the dosage of parasites increased, a greater proportion of parasites was found in the adult male relative to males at lower dosages, while the proportion of parasites found in the adult female did not differ across doses (Table 3, Table F in S1 File). Furthermore, neither wing length nor time to emergence was related to the proportion of oocysts remaining in the adult (Table 3; S1 and S2 Figs).

**Table 3:** ANCOVA results for the response variable percentage of oocysts in the adult and the explanatory variables of sex and parasite dosage, with covariates of wing length and time to emergence, and a random effect for microcosm.

Effect	D.f.	Partial η^2^	χ^2^	P
Wing Length	1	0.01	1.48	0.22
Time to Emergence	1	0.00	0.34	0.56
Dosage	2	0.11	7.05	0.03*
Sex	1	0.01	0.59	0.44
Dosage:Sex	2	0.12	11.39	<0.01*
Residuals	88			

* Significant at a 0.05 alpha level.

## Discussion

These results support our hypothesis that *A*. *taiwanensis* uses different dispersal strategies based on host sex. Moreover, in male mosquitoes, dispersal method depends upon parasite exposure levels, with higher parasite exposure levels corresponding to an increase in parasite investment in the adult male. Thus, *A*. *tawianensis* appears to exhibit a mixture of dispersal strategies due both to “environment” (that is, host sex) and density of conspecifics (that is, dosage of parasite). For the most part, our results are consistent with those of Fellous and Koella [15], in that we see a mixed dispersal strategy that varied between males and females. However, we observed a response to density only in parasites infecting male mosquitoes, whereas Fellous and Koella [15] saw a density effect of *A*. *culicis* infecting both male and female *Ae*. *aegypti*. This could be due to differences in experimental design, as our two highest doses were beyond those used by Fellous and Koella, or it could be due to differences between *A*. *taiwanensis* and *A*. *culicis* in how they respond to intraspecific density, as well as to intrinsic differences between the hosts, *Ae*. *aegypti* and *Ae*. *albopictus*.

As there was no relationship between the body size or time to emergence of mosquitoes and the proportion of oocysts remaining in the adult following emergence, we reject the hypothesis that the faster male development or the smaller body size of males are somehow responsible for a different dispersal method of the parasite in male mosquitoes compared to female mosquitoes. If the mixed dispersal methods we observed in *A*. *taiwanensis* within *Ae*. *albopictus* are instead a result of adaptation to its native host, as was proposed in Fellous and Koella [15], then we might expect that this phenomenon would be absent in non-native hosts. In a previous study, in which we found life cycle completion of *A*. *taiwanensis* in a non-native host (*Aedes japonicus*), we found no difference in presence or absence of oocysts between pupal exuviae and adult homogenate based on sex [28]. Moreover, Tseng [47] demonstrated that *A*. *taiwanensis* produces fewer oocysts when infecting F2 hybrid populations of *Ae*. *albopictus* than either parental strain, suggesting some degree of adaptation within populations of coevolving *Ae*. *albopictus* and *A*. *taiwanensis*. These results, combined with previous work in *Ascogregarina*, and our present failure to detect an effect of male size or time to emergence of mosquitoes on the percentage of oocysts in the adult, suggest an adaptation to female hosts, as opposed to physical limitations. However, as adult males do not return to larval habitats, what adaptive value a density response in males might hold is unknown.

In response to increasing density of parasites, we saw a steep decline in both measures of parasite growth rate at parasite doses above 100/ml. Thus, we provide the first evidence of a response to intraspecific density in *A*. *taiwanensis*. This response may be classical density dependence due to resource limitation, or it could result from activating the mosquito immune system; regardless of the exact mechanism, it is evidence that *Ascogregarina* parasites within *Aedes* mosquitoes have reduced growth in hosts that are heavily parasitized, even under optimal food conditions. This decline was paired with a simultaneous increase in the proportion of oocysts allocated to the adult male, consistent with the hypothesis that this parasite would shift dispersal methods in a density-dependent manner, yet the total number of oocysts in males and females did not differ between dosages. Our observation that this shift in dispersal occurred only in male mosquitoes may be due to our choice of oocyst dosages, which may have been high enough to have already induced dispersal in parasites infecting female mosquitoes. It is largely unknown what a natural dosage of this parasite might be. Natural exposure to oocysts can likely be quite high; each oocyst will release eight sporozoites, and late instar larvae have been reported to contain hundreds to more than two thousand trophozoites in field collections [29], but direct comparisons from such field observations would fail to account for mosquito immune system and likely undervalue initial exposure. Moreover, *Ascogregarina* prevalence in *Aedes* mosquitoes varies seasonally [36] and thus we would expect oocyst exposure levels to vary as well; in our own observations, we have found several treehole habitats to contain oocyst densities similar to our two lowest parasite dosages, with oocyst values below 100/ml early in the season and in large containers, while oocyst values were between 500 and 1000/ml later in the season (unpublished data). Thus, it is possible that our highest dosage of oocysts is beyond realistic field conditions; however, even an unrealistic density of oocysts can provide insight into density and crowding in this host/parasite system.

Our study provides substantial support that *A*. *taiwanensis* is a parasite, even under the low levels of intraspecific larval competition with high resource availability. We found a significant decline in average wing size for infected mosquitoes, both male and female. Further, we found a significant effect of the parasite on mortality and our proxy of per capita growth rate, r’. It is likely that our r’ estimates are a lower bound on the effect of the parasite; Comiskey et al. [36] found that fecundity in *Ae*. *albopictus* declined due to exposure to *A*. *taiwanensis*. Consistent with our study, they also found a reduction in wing size, but did not determine to what degree the decrease in fecundity was due to the body size reduction they observed. However, the relative reduction in fecundity of infected mosquitoes compared to controls was greater than the relative loss of body size, and as such, it is likely that *A*. *taiwanensis* would further reduce the fitness of its hosts beyond what we have demonstrated.

While previous studies demonstrate that host environment can alter host responses to parasitism in a sex-specific manner [37,48], we show here that a parasite can alter its response to its environment in a manner that is specific to host sex. While it may seem counteradaptive for a parasite at high densities to favor adult male mosquito hosts, it is possible the parasite alters male behavior in ways not yet described, and as such the effect of the parasite on adults should be further studied. As our study focused exclusively on the consequences of parasite infection during the larval and pupal stage, future studies could incorporate parameters related to adult longevity or dispersal, along with assessing whether the parasite has any effect on male mosquitoes that has not yet been described, such as suicidal drowning or post-emergence mortality within the larval habitat.

## Supporting information

S1 Fig The percentage of oocysts in the adult, plotted by the wing length of adult, the sex of the mosquito (rows), and the dosage of parasites (columns). There was no relationship between the body size of mosquitoes and proportion of oocysts remaining in the adult; however, there was a significant effect of sex.
(TIF)

S2 Fig The percentage of oocysts in the adult, plotted by the time to emergence of the mosquito, the sex of the mosquito (rows), and the dosage of parasites (columns). There was no relationship between time to emergence and proportion of oocysts remaining in the adult, though sex had a significant effect on time to emergence.
(TIF)

S1 File Supplemental tables A through G are attached as supplemental material. (DOCX)

## References

[1] Travis JMJ, Murrell DJ, Dytham C. The evolution of density-dependent dispersal. Proc R Soc B Biol Sci. 1999;266: 1837 doi: 10.1098/rspb.1999.0854

[2] Matthysen E. Density-dependent dispersal in birds and mammals. Ecography. 2005;28: 403–416. doi: 10.1111/j.0906-7590.2005.04073.x

[3] Ronce O. How Does It Feel to Be like a Rolling Stone? Ten Questions about Dispersal Evolution. Annu Rev Ecol Evol Syst. 2007;38: 231–253.

[4] Friedenberg NA. Experimental evolution of dispersal in spatiotemporally variable microcosms. Ecol Lett. 2003;6: 953–959. doi: 10.1046/j.1461-0248.2003.00524.x

[5] Järvinen O, Vepsäläinen K. Wing dimorphism as an adaptive strategy in water-striders (Gerris). Hereditas. 1976;84: 61–68. doi: 10.1111/j.1601-5223.1976.tb01196.x

[6] Landin J, Vepsäläinen K. Spring Dispersal Flights of Pond-Skaters Gerris spp. (Heteroptera). Oikos. 1977;29: 156–160. doi: 10.2307/3543307

[7] Vepsäläinen K. Wing Dimorphism and Diapause in Gerris: Determination and Adaptive Significance In: Dingle H, editor. Evolution of Insect Migration and Diapause. Springer US; 1978 pp. 218–253. Available: http://link.springer.com/chapter/10.1007/978-1-4615-6941-1_10

[8] Stonedahl GM, Lattin JD. Gerridae or water striders of Oregon and Washington (Hemiptera:Heteroptera). 1982; Available: http://agris.fao.org/agris-search/search.do?recordID=US201300672906

[9] Muraji M, Nakasuji F. Comparative studies on life history traits of three wing dimorphic water bugs,Microvelia spp. Westwood (Heteroptera: Veliidae). Res Popul Ecol. 1988;30: 315–327. doi: 10.1007/BF02513252

[10] Zera AJ, Denno RF. Physiology and Ecology of Dispersal Polymorphism in Insects. Annu Rev Entomol. 1997;42: 207–230. doi: 10.1146/annurev.ento.42.1.207 15012313 10.1146/annurev.ento.42.1.207

[11] Denno RF, Perfect JR. Planthoppers: Their Ecology and Management. Springer Science & Business Media; 2012.

[12] Denno RF, Roderick GK, Olmstead KL, Dobel HG. Density-Related Migration in Planthoppers (Homoptera: Delphacidae): The Role of Habitat Persistence. Am Nat. 1991;138: 1513–1541.

[13] Andreadis TG, Simakova AV, Vossbrinck CR, Shepard JJ, Yurchenko YA. Ultrastructural characterization and comparative phylogenetic analysis of new microsporidia from Siberian mosquitoes: evidence for coevolution and host switching. J Invertebr Pathol. 2012;109: 59–75. doi: 10.1016/j.jip.2011.09.011 22001630 10.1016/j.jip.2011.09.011

[14] Andreadis TG, Hall DW. Development, ultrastructure, and mode of transmission of Amblyospora sp. (Microspora) in the mosquito. J Protozool. 1979;26: 444–452. 536933 10.1111/j.1550-7408.1979.tb04651.x

[15] Fellous S, Koella JC. Different transmission strategies of a parasite in male and female hosts. J Evol Biol. 2009;22: 582–588. doi: 10.1111/j.1420-9101.2008.01665.x 19210596 10.1111/j.1420-9101.2008.01665.x

[16] Ahmad R, Ismail A, Saat Z, Lim LH. Detection of dengue virus from field Aedes aegypti and Aedes albopictus adults and larvae. Southeast Asian J Trop Med Public Health. 1997;28: 138–142. 9322296

[17] Bodenmann P, Genton B. Chikungunya: an epidemic in real time. Lancet. 2006;368: 258 doi: 10.1016/S0140-6736(06)69046-6 16844497 10.1016/S0140-6736(06)69046-6

[18] Grard G, Caron M, Mombo IM, Nkoghe D, Mboui Ondo S, Jiolle D, et al Zika Virus in Gabon (Central Africa)– 2007: A New Threat from Aedes albopictus? PLoS Negl Trop Dis. 2014;8 doi: 10.1371/journal.pntd.0002681 24516683 10.1371/journal.pntd.0002681 PMC3916288

[19] Barker CM, Paulson SL, Cantrell S, Davis BS. Habitat Preferences and Phenology of Ochlerotatus triseriatus and Aedes albopictus (Diptera: Culicidae) in Southwestern Virginia. J Med Entomol. 2003;40: 403–410. doi: 10.1603/0022-2585-40.4.403 14680103 10.1603/0022-2585-40.4.403

[20] Chen WJ. The Life Cycle of Ascogregarina taiwanensis (Apicomplexa:Lecudinidae). Parasitol Today. 1999;15: 153–156. 10322337 10.1016/s0169-4758(99)01418-0

[21] Bonizzoni M, Gasperi G, Chen X, James AA. The invasive mosquito species Aedes albopictus: current knowledge and future perspectives. Trends Parasitol. 2013;29: 460–468. doi: 10.1016/j.pt.2013.07.003 23916878 10.1016/j.pt.2013.07.003 PMC3777778

[22] Munstermann LE, Wesson DM. First Record of Ascogregarina Taiwanensis (Apicomplexa: Lecudinidae) in North American Aedes Albopictus. J Am Mosq Control Agency. 1990;6: 235–243. 2370530

[23] Chen WJ, Yang CH. Developmental synchrony of Ascogregarina taiwanensis (Apicomplexa: Lecudinidae) within Aedes albopictus (Diptera: Cuclicidae). J Med Entomol. 1996;33: 212–215. 8742523 10.1093/jmedent/33.2.212

[24] Chen W-J, Chia-yi C, Wu S. Ultrastructure of Infection, Development and Gametocyst Formation of *Ascogregarina taiwanensis* (Apicomplexa: Lecudinidae) in Its Mosquito Host, *Aedes albopictus* (Diptera: Culicidae). J Eurkaryotic Microbiol. 1997;44: 101–108. doi: 10.1111/j.1550-7408.1997.tb05945.x 10.1111/j.1550-7408.1997.tb05945.x 9190261

[25] Chen W-J, Wu S-T, Chow C-Y, Yang C-H. Sporogonic Development of the Gregarine Ascogregarina taiwanensis (Lien and Levine) (Apicomplexa: Lecudinidae) in Its Natural Host Aedes albopictus (Skuse) (Diptera: Culicidae). J Eurkaryotic Microbiol. 1997;44: 326–331. doi: 10.1111/j.1550-7408.1997.tb05674.x

[26] Aliabadi BW, Juliano SA. Escape from gregarine parasites affects the competitive interactions of an invasive mosquito. Biol Invasions. 2002;4: 283–297. doi: 10.1023/A:1020933705556 19777120 10.1023/A:1020933705556 PMC2748405

[27] Reyes-Villanueva F, Becnel JJ, Butler JF. Susceptibility of Aedes aegypti and Aedes albopictus larvae to Ascogregarina culicis and Ascogregarina taiwanensis (Apicomplexa: Lecudinidae) from Florida. J Inverebrate Pathol. 2003;84: 47–53. 10.1016/s0022-2011(03)00119-8 13678712

[28] Erthal JA, Soghigian JS, Livdahl T. Life Cycle Completion of Parasite Ascogregarina taiwanensis (Apicomplexa: Lecudinidae) in Non-Native Host Ochlerotatus japonicus (Diptera: Culicidae). J Med Entomol. 2012;49: 1109–1117. doi: 10.1603/ME12018 23025193 10.1603/me12018

[29] Beier JC, Craig GB. Gregarine Parasites of Mosquitoes. Integrated mosquito control methodologies. 1985.

[30] Passos RA dos Tadei WP. Parasitism of Ascogregarina taiwanensis and Ascogregarina culicis (Apicomplexa: Lecudinidae) in larvae of Aedes albopictus and Aedes aegypti (Diptera: Culicidae) from Manaus, Amazon region, Brazil. J Invertebr Pathol. 2008;97: 230–236. doi: 10.1016/j.jip.2007.09.008 18028941 10.1016/j.jip.2007.09.008

[31] Kaplan L, Kendell D, Robertson D, Livdahl T, Khatchikian C. Aedes aegypti and Aedes albopictus in Bermuda: extinction, invasion, invasion and extinction. Biol Invasions. 2010;

[32] Roychoudhury S, Kobayashi M. New Findings on the Developmental Process of Ascogregarina Taiwanensis and Ascogregarina Culicis in Aedes Albopictus and Aedes Aegypti. J Am Mosq Control Agency. 2006;22: 29–36. 10.2987/8756-971X(2006)22[29:NFOTDP]2.0.CO;2 16646318

[33] Tseng M. Ascogregarine parasites as possible biocontrol agents of mosquitoes. J Am Mosq Control Agency. 2007;23: 30–34. 10.2987/8756-971X(2007)23[30:APAPBA]2.0.CO;2 17853595

[34] Soghigian J, Valsdottir LR, Livdahl TP. A parasite’s modification of host behavior reduces predation on its host. Ecol Evol. 2017;7: 1453–1461. doi: 10.1002/ece3.2748 28261457 10.1002/ece3.2748 PMC5330890

[35] Garcia JJ, Fukuda T, Becnel JJ. Seasonality, Prevalence, and Pathogenicity of the gregarine Ascogregarina Taiwanensis (Apicomplexa: Lecudinidae) is Mosquitoes from Florida. J Am Mosq Control Agency. 1994;10: 413–418. 7807086

[36] Comiskey NM, Lowrie RC, Wesson DM. Effect of nutrient levels and Ascogregarina taiwanensis (Apicomplexa: Lecudinidae) infections on the vector competence of Aedes albopictus (Diptera: Culicidae) for Dirofilaria immitis (Filarioidea: Onchocercidae). J Med Entomol. 1999;36: 55–61. 10071493 10.1093/jmedent/36.1.55

[37] Tseng M. Sex-specific response of a mosquito to parasite and crowding. Proc R Soc B Biol Sci. 2004;271: S186–S188. 10.1098/rsbl.2003.0139 PMC1810031 15252979

[38] Livdahl TP, Sugihara G. Non-Linear Interactions of Populations and the Importance of Estimating Per Capita Rates of Change. J Anim Ecol. 1984;53: 573–580. doi: 10.2307/4535

[39] O’Neal PA, Juliano SA. Seasonal variation in competition and coexistence of Aedes mosquitoes: stabilizing effects of egg mortality or equalizing effects of resources? J Anim Ecol. 2013;82. 10.1111/j.1365-2656.2012.02017.x PMC3480969 22823120

[40] Lounibos LP, Suárez S, Menéndez Z, Nishimura N, Escher RL, O’Connell SM, et al Does temperature affect the outcome of larval competition between Aedes aegypti and Aedes albopictus? J Vector Ecol J Soc Vector Ecol. 2002;27: 86–95. 12125878

[41] R Core Team. R: A language and environment for statistical computing. [Internet]. R Foundation for Statistical Computing, Vienna, Austria.; 2015. Available: http://www.R-project.org/.

[42] Fox J, Weisberg S. An R Companion to Applied Regression. SAGE Publications; 2011.

[43] Mitchell A, Bergmann PJ. Thermal and moisture habitat preferences do not maximize jumping performance in frogs. Funct Ecol. 2015; 733–742. doi: 10.1111/1365-2435.12535

[44] Navarro D. Learning statistics with R: A tutorial for psychology students and other beginners. (Version 0.5) [Internet]. Adelaide, Australia: University of Adelaide; 2015. Available: http://ua.edu.au/ccs/teaching/lsr

[45] Bates D, Machler M, Bolker B, Walker S. Fitting Linear Mixed-Effects Models Using lme4. J Stat Softw. 2015;67 doi: 10.18637/jss.v067.i01

[46] Lenth RV. Least-Squares Means: The R Package lsmeans. J Stat Softw. 2016;69: 1–33. doi: 10.18637/jss.v069.i01

[47] Tseng M. The Effect of Parasitism and Interpopulation Hybridization on Aedes albopictus (Diptera: Culicidae) Fitness. J Med Entomol. 2017; doi: 10.1093/jme/tjx075 28419266 10.1093/jme/tjx075

[48] Sheridan LAD, Poulin R, Ward DF, Zuk M. Sex differences in parasitic infections among arthropod hosts: is there a male bias? Oikos. 2000;88: 327–334. doi: 10.1034/j.1600-0706.2000.880211.x

