# Supplementary figures and images for: Differential response to mosquito host sex and parasite dosage suggest mixed dispersal strategies in the parasite Ascogregarina taiwanensis
Source: PLoS One. 2017 Sep 13;12(9):e0184573. doi: 10.1371/journal.pone.0184573 (PMC5597222; doi:10.1371/journal.pone.0184573)

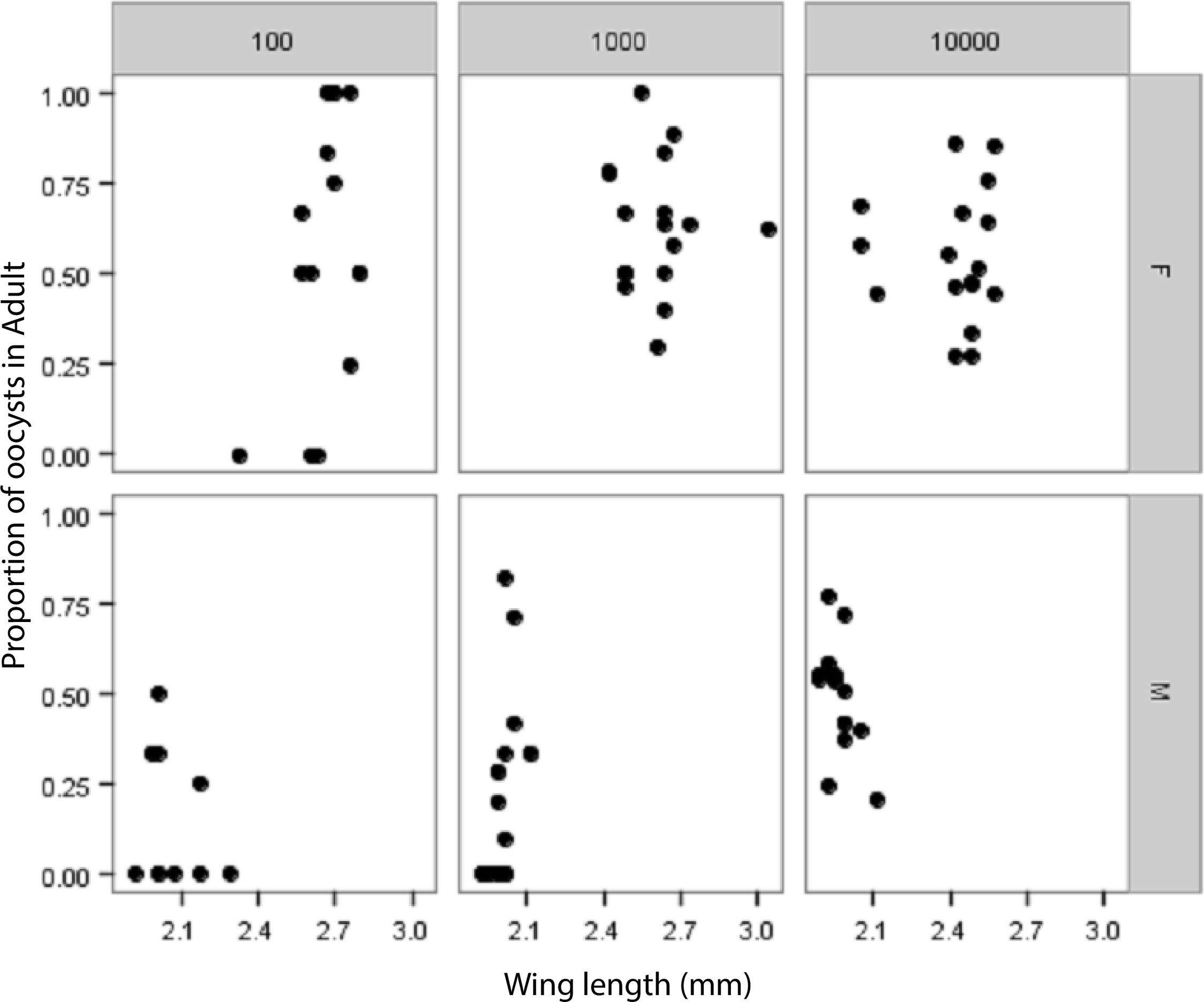

Supplement: S1 Fig — There was no relationship between the body size of mosquitoes and proportion of oocysts remaining in the adult; however, there was a significant effect of sex. (TIF) [file pone.0184573.s001.tif]

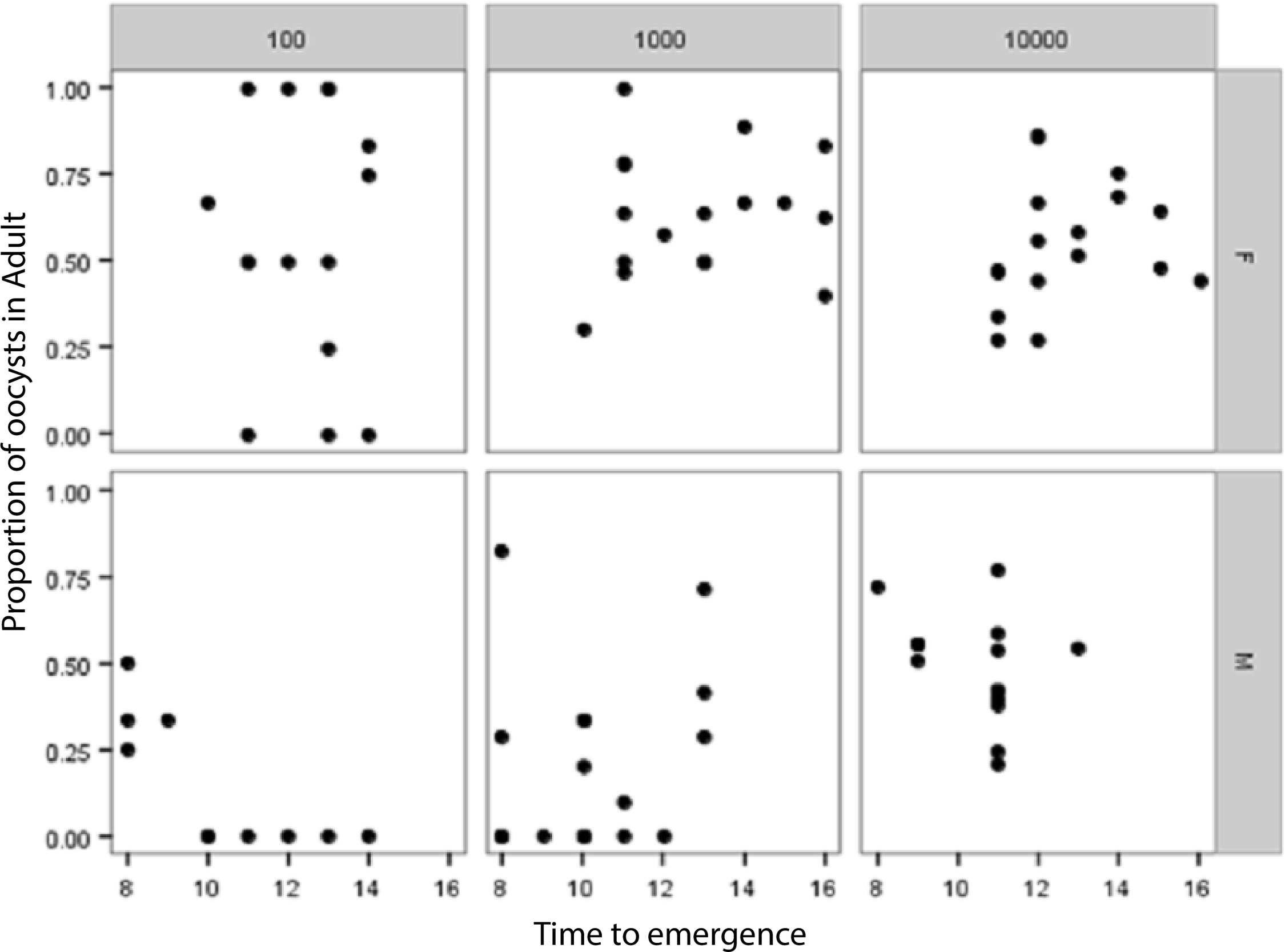

Supplement: S2 Fig — There was no relationship between time to emergence and proportion of oocysts remaining in the adult, though sex had a significant effect on time to emergence. (TIF) [file pone.0184573.s002.tif]
