# Supplementary material for: Differential response to mosquito host sex and parasite dosage suggest mixed dispersal strategies in the parasite Ascogregarina taiwanensis
Source: PLoS One. 2017 Sep 13;12(9):e0184573. doi: 10.1371/journal.pone.0184573 (PMC5597222; doi:10.1371/journal.pone.0184573)
Supplement: S1 File — (DOCX) [file pone.0184573.s003.docx]

SUPPLEMENTARY TABLES

| Supplementary Table A: Full ANOVA Models for various response variables, and the explanatory variable of dosage | | | | |
| --- | --- | --- | --- | --- |
| Per Capita Growth Rate, r' | |  |  |  |
|  | Sum Sq | Df | F-value | Pr(>F) |
| Dosage | 0.002 | 3 | 9.071 | 0.005929 |
| Residuals | 0.000588 | 8 |  |  |
| Proportion of hosts surviving in microcosms | | | | |
|  | Sum Sq | Df | F-value | Pr(>F) |
| Dosage | 0.128519 | 3 | 9.6389 | 0.004933 |
| Residuals | 0.035556 | 8 |  |  |
| Mean Sex Ratio |  |  |  |  |
|  | Sum Sq | Df | F-value | Pr(>F) |
| Dosage | 0.002028 | 3 | 0.0396 | 0.9887 |
| Residuals | 0.126737 | 8 |  |  |
|  |  |  |  |  |
| Parasite per capita growth rate (rp) | | | | |
|  | Sum Sq | Df | F-value | Pr(>F) |
| Dosage | 0.003339 | 2 | 27.11 | 0.000989 |
| Residuals | 0.000370 | 6 |  |  |
|  |  |  |  |  |
| Parasite per capita growth rate (rm) | | | | |
|  | Sum Sq | Df | F-value | Pr(>F) |
| Dosage | 0.004768 | 2 | 22.75 | 0.00158 |
| Residuals | 0.000629 | 6 |  |  |

| Supplementary Table B: Randomization ANOVA on time to emergence with explanatory variables dosage and sex. | | | | | | |
| --- | --- | --- | --- | --- | --- | --- |
|  | Df | Sum Sq | Mean Sq | F value | Pr(>F) | Prand |
| Dosage | 2 | 1.858 | 0.9292 | 0.3171 | 7.29E-01 | 0.732 |
| sex | 1 | 133.042 | 133.0417 | 45.4017 | 8.87E-10 | 0.00 |
| Dosage:Sex | 2 | 5.974 | 2.987 | 1.0193 | 3.64E-01 | 0.346 |
| Residuals | 105 | 307.684 | 2.9303 | NA | NA | NA |

| Supplementary Table C: Randomization ANOVA results for the response variable total oocysts in the mosquito and the explanatory variables of sex and parasite dosage. | | | | | |  |  |
| --- | --- | --- | --- | --- | --- | --- | --- |
| Response Variable | D.f. | Partial η^2^ | F | P** |  |  |  |
| Wing Length | 1 | 0.01 | 1.36 | 0.22 |  |  |  |
| Time to Emergence | 1 | 0.01 | 1.39 | 0.22 |  |  |  |
| Dosage | 2 | 0.81 | 290.16 | <0.01* |  |  |  |
| Sex | 1 | 0.00 | 0.00 | 0.99 |  |  |  |
| Dosage:Sex | 2 | 0.03 | 1.55 | 0.21 |  |  |  |
| Residuals | 103 |  |  |  |  |  |  |
| * Significant at a 0.05 alpha level.  ** P-Value from randomization test. |  |  |  |  |  | |  |

| Supplementary Table D: Tukey’s HSD pairwise comparison of the proportion of host mosquitoes surviving at different dosages (oocysts/ml). | | | | |
| --- | --- | --- | --- | --- |
| Comparison | Difference | L. Bound | U. Bound | P-Value |
| 100-0 | -0.04444 | -0.21876 | 0.12987 | 0.845201 |
| 1000-0 | -0.15556 | -0.32987 | 0.018758 | 0.081322 |
| 10000-0 | -0.26667 | -0.44098 | -0.09235 | **0.005234** |
| 1000-100 | -0.11111 | -0.28543 | 0.063203 | 0.250278 |
| 10000-100 | -0.22222 | -0.39654 | -0.04791 | **0.014916** |
| 10000-1000 | -0.11111 | -0.28543 | 0.063203 | 0.250278 |

| Supplementary Table E: Tukey’s HSD pairwise comparison of the per capita growth rate for the host , r’, at different dosages (oocysts/ml). | | | | | |
| --- | --- | --- | --- | --- | --- |
| Comparison | Difference | L. Bound | U. Bound | | P-Value |
| 100-0 | -0.00975 | -0.03415 | 0.014657 | 0.599388 | |
| 1000-0 | -0.02538 | -0.04978 | -0.00098 | **0.041766** | |
| 10000-0 | -0.03176 | -0.05616 | -0.00735 | **0.013332** | |
| 1000-100 | -0.01563 | -0.04004 | 0.00877 | 0.246958 | |
| 10000-100 | -0.02201 | -0.04641 | 0.002396 | 0.07792 | |
| 10000-1000 | -0.00637 | -0.03078 | 0.018029 | 0.835968 | |

| Supplementary Table F: Tukey’s HSD pairwise comparison between the percent of oocysts at different dosages and sexes of host mosquitoes. | | | | |
| --- | --- | --- | --- | --- |
| Comparison | Difference | L. Bound | U. Bound | P-Value |
| 1000:F-100:F | 0.054283 | -0.17298 | 0.281547 | 0.98201 |
| 10000:F-100:F | -0.03447 | -0.26486 | 0.195916 | 0.997948 |
| 100:M-100:F | -0.46496 | -0.72403 | -0.20589 | **1.62E-05** |
| 1000:M-100:F | -0.38853 | -0.61296 | -0.1641 | **3.45E-05** |
| 10000:M-100:F | -0.09656 | -0.33427 | 0.141162 | 0.843922 |
| 10000:F-1000:F | -0.08875 | -0.31245 | 0.134943 | 0.856492 |
| 100:M-1000:F | -0.51924 | -0.77238 | -0.26611 | **7E-07** |
| 1000:M-1000:F | -0.44281 | -0.66037 | -0.22525 | **8E-07** |
| 10000:M-1000:F | -0.15084 | -0.38208 | 0.080401 | 0.409213 |
| 100:M-10000:F | -0.43049 | -0.68643 | -0.17455 | **6.12E-05** |
| 1000:M-10000:F | -0.35406 | -0.57488 | -0.13324 | **0.00015** |
| 10000:M-10000:F | -0.06208 | -0.2964 | 0.172227 | 0.971606 |
| 1000:M-100:M | 0.076434 | -0.17416 | 0.32703 | 0.948426 |
| 10000:M-100:M | 0.368406 | 0.105844 | 0.630968 | **0.001301** |
| 10000:M-1000:M | 0.291972 | 0.063515 | 0.520429 | **0.004514** |

| Supplementary Table G: Tukey’s HSD pairwise comparison between different parasite doses, for each measure of parasite growth | | | | |
| --- | --- | --- | --- | --- |
| r_p_ – Including all male oocysts | | | | |
| Comparison | Difference | L. Bound | U. Bound | P-Value |
| 1000-100 | -0.04527 | -0.06493 | -0.02561 | **0.000983** |
| 10000-100 | -0.03414 | -0.0538 | -0.01448 | **0.004284** |
| 10000-1000 | 0.011124 | -0.00854 | 0.030784 | 0.26805 |
| r_m_ – Excluding oocysts from adult males | | | | |
| Comparison | Difference | L. Bound | U. Bound | P-Value |
| 1000-100 | -0.04972 | -0.07536 | -0.02407 | **0.002444** |
| 10000-100 | -0.04788 | -0.07353 | -0.02224 | **0.002966** |
| 10000-1000 | 0.001834 | -0.02381 | 0.027479 | 0.973909 |
